# *Heterorhabditis bacteriophora* symbiotic and axenic nematodes modify the *Drosophila melanogaster* larval microbiome

**DOI:** 10.3389/fmicb.2025.1598221

**Published:** 2025-06-18

**Authors:** Sreeradha Mallick, Christina Pavloudi, Jimmy Saw, Ioannis Eleftherianos

**Affiliations:** ^1^Department of Biological Sciences, The George Washington University, Washington, DC, United States; ^2^European Marine Biological Resource Centre-European Research Infrastructure Consortium (EMBRC-ERIC), Paris, France

**Keywords:** insects, innate immunity, *Drosophila*, entomopathogenic nematodes, microbiome, host-pathogen interactions

## Abstract

The *Drosophila melanogaster* microbiome is crucial for regulating physiological processes, including immune system development and function. *D. melanogaster* offers distinct advantages over vertebrate models, allowing a detailed investigation of host-microbiota interactions and their effects on modulating host defense systems. It is an outstanding model for studying innate immune responses against parasites. Entomopathogenic nematodes (EPNs) activate immune signaling in the fly, leading to immune responses to combat infection. However, the impact of EPN infection on the host larval microbiome remains poorly understood. Therefore, we investigated whether EPN infection affects the *D. melanogaster* larval microbiome. We infected third-instar *D. melanogaster* larvae with *Heterorhabditis bacteriophora* symbiotic nematodes (containing *Photorhabdus luminescens* bacteria) and axenic nematodes (devoid of symbiotic bacteria). *Drosophila melanogaster* microbiome analysis revealed statistically significant differences in microbiome composition between uninfected and EPN-infected larvae. Notably, infection with axenic nematodes resulted in 68 unique species, causing a significant shift in the *D. melanogaster* larval microbiome and an increase in bacterial diversity compared to larvae infected with symbiotic nematodes. This suggests that the absence of the endosymbiont creates ecological niches for unique species and a more diverse microbiome in larvae infected with the axenic nematodes. This research will enhance our understanding of microbial species within the *D. melanogaster* microbiome that regulate homeostasis during nematode infection. These insights could be beneficial in developing innovative strategies for managing agricultural pests and disease vectors.

## Introduction

Microbial communities within multicellular organisms consist of bacteria, protozoa, viruses, and fungi, which often exist as commensals. These communities coexist with their hosts and play crucial roles in regulating various physiological processes. They also contribute to the modulation of the host immune response. In return, the host immune system has developed various strategies to maintain a mutualistic relationship with the microbiome (Belkaid and Harrison, 2017). A balanced microbiome is vital for a healthy life, as any shifts in its composition can lead to potential health issues. Approximately 70–80% of immune-related cells are found in the gut, creating a complex relationship between the immune system and the gut microbiome (Wiertsema et al., 2021). The gut lining controls defense mechanisms that recognize invading parasites which leads to the activation of immune responses. Previous studies have shown that biological mechanisms such as intestinal epithelial barrier and renewal, immune system responses, and gut motility involved in gut homeostasis are highly conserved between the fruit fly, *Drosophila melanogaster*, and vertebrates. Although some distinctions exist between the gut of humans and *Drosophila*, several essential pathways related to metabolism and immunity that promote gut health are conserved in both organisms (Capo et al., 2019). Additionally, the genome sequence of *D. melanogaster* reveals that over 75% of human disease-related genes have orthologs in the fruit fly (Reiter et al., 2001).

*Drosophila melanogaster* depends on several innate immune mechanisms to battle parasitic infections that resemble those in vertebrates. Major immune signaling pathways and transcriptional regulators found in mammalian species are evolutionarily conserved in fruit flies. This makes *D. melanogaster* a preferred model organism for studying the regulation of innate immune signaling pathways upon pathogenic infection (Lemaitre and Hoffmann, 2007). *Drosophila melanogaster* is also an excellent model in microbiome research due to the availability of multiple genetic and genomic tools that allow researchers to answer a wide variety of biological questions, which include the host’s interactions with its microbial communities, factors affecting the microbiome composition, and how the interactions impact host’s health and development (Douglas, 2018). Recent work on entomopathogenic nematodes (EPNs) and their interactions with insect hosts has focused on identifying and characterizing the function of EPN infection factors. EPNs can quickly invade insects and interfere with their innate immune system during the early stages of infection. A key stage of the EPN life cycle is the infective juvenile (IJ), where the nematodes rely on the presence of a suitable insect host to support their survival and promote their growth and development. During this stage, the IJs locate, invade the insect host, and utilize its resources to complete their life cycle (Tarasco et al., 2023). Following insect infection, the IJs mature into adults, which then lay eggs that go through four larval stages (J1 to J4) to produce the next generation of adults (Noguez et al., 2012).

The EPN *Heterorhabditis bacteriophora* maintains a mutualistic relationship with the Gram-negative bacteria *Photorhabdus luminescens* (Waterfield et al., 2009). The bacterial cells occupy the anterior region of the IJ intestine (McLean et al., 2018). *Heterorhabditis bacteriophora* nematodes and their *P. luminescens* mutualistic bacteria are able to modulate the insect immune response by modifying the activity of innate immune signaling pathways during infection (Eleftherianos et al., 2010; Ozakman and Eleftherianos, 2021). This allows the nematode-bacterial complex to evade or suppress insect host defenses. EPN immunomodulation strategies increase the virulence of the parasites and contribute to faster insect death (Bastin and Eleftherianos, 2023; Kenney et al., 2021; Ozakman et al., 2021). Although the mutualistic *P. luminescens* bacteria contribute to EPN pathogenicity, *H. bacteriophora* nematodes lacking their bacterial partners, referred to as axenic nematodes, are still pathogenic to insects (Castillo et al., 2012).

Knowledge of the interaction between the host microbiome and invading pathogens is crucial for uncovering the function of microorganisms that participate in gut immune processes. Although microbial communities are known to influence the host’s physiology, their mechanistic role in the innate immune response to parasitic nematodes remains poorly understood. In this study, we exposed wild-type *D. melanogaster* larvae to *H. bacteriophora* symbiotic or axenic nematodes. The hypothesis of the study is that EPN infection alters the structure and composition of the insect microbiome regardless of the presence or absence of their mutualistic bacteria. Exploring whether and how parasitic nematode infection impacts the host microbiome can provide insights into developing novel approaches for the efficient management of destructive insect pests and disease vectors.

## Materials and methods

### Fly stock maintenance

The *D. melanogaster* stocks were maintained and amplified using a standard diet (Fly Food B, LabExpress, Ann Arbor, MI). The diet was supplemented with yeast (Carolina Biological Supply, Burlington, NC). Third-instar larvae from the *D. melanogaster w^1118^* wild-type line were used in the experiments. The flies were kept in an incubator at 25°C and a 12-h light: dark photoperiod.

### Nematode stocks

Symbiotic and axenic *H. bacteriophora* TT01 nematodes were used in the experiments. The nematode stocks have been maintained and amplified in the Eleftherianos lab for several years. For nematode amplification, final stage larvae of the greater wax moth, *Galleria mellonella*, were infected with *H. bacteriophora* infective juveniles on a 6 cm Petri dish. The insect larvae were maintained for a week in an incubator set at 25°C and a 12-h light: dark photoperiod. Then, the infected caterpillars were transferred to water traps, as described before (Heryanto et al., 2022). Fourteen days later, the new generation of infective juveniles was collected in a 50 mL flask, maintained in an incubator at 28°C, and closely monitored under a stereoscope (Tritech Research, CA).

### Fly larval infection with entomopathogenic nematodes

Nematode infection experiments of *D. melanogaster w^1118^* larvae were performed using 96-well microplates (ThermoFisher Scientific, USA). First, each well was loaded with 100 μL of 1.25% agarose gel (Fisher Scientific, USA). Then, single larvae were transferred with a fine paintbrush to individual wells of the 96-well microplate. Approximately 100 infective juveniles of either symbiotic or axenic *H. bacteriophora* suspended in 10 μL of sterile water were added to each larva (20 larvae were infected per survival experiment). Following infection, the microplates were sealed with Masterclear real-time PCR film (Eppendorf, Enfield, CT) and ventilated by creating small holes. Larvae that escaped from the well were excluded from this calculation. Control experiments involved treatment of *w^1118^* larvae with 10 μL of sterile water only (uninfected controls). Three independent experiments with new batches of *D. melanogaster* larvae and *H. bacteriophora* symbiotic or axenic nematodes were performed on different days.

### DNA extraction

Nematode-infected and uninfected *D. melanogaster* larvae were collected at 24- and 48-h time points. Only live larvae were collected in 1.5 mL Eppendorf tubes and stored in a freezer at −80°C for further processing. Each sample involved 100 larvae. DNA extraction was performed using the Qiagen DNA extraction kit protocol (Qiagen, Germantown, MD). DNA concentrations for each sample were measured with the Qubit 4 Fluorometer (Thermo Fisher Scientific, Waltham, MA). Each DNA sample had a minimum concentration of 10 ng/μL in a total volume of 100 μL. The purity of the samples ranged from 1.45 to 1.91, and the DNA concentrations varied between 20 ng/μL and 100 ng/μL.

### Library preparation and high-throughput sequencing

Amplicon libraries were prepared using Zymo Research’s Quick-16S kit with phased primers (341F – 806R) targeting the V3-V4 regions of the 16S rRNA gene. Following clean up and normalization, samples were sequenced on a P1 600cyc NextSeq2000 Flowcell to generate 2 × 301 bp paired end (PE) reads. Quality control and adapter trimming was performed with bcl-convert1 (v4.2.4).

All the raw sequence files of this study were submitted to the European Nucleotide Archive (ENA) (O'Cathail et al., 2025) with the study accession number PRJEB85826 (available at http://www.ebi.ac.uk/ena/data/view/ PRJEB85826).

### Amplicon sequence analysis

DADA2 was used to filter and trim sequences, infer amplicon sequence variants (ASVs) and remove sequencing errors and chimeric sequences. Taxonomy assignment was performed using SILVA release 138.2 (Quast et al., 2013). ASVs belonging to Mitochondria and Chloroplasts were removed before proceeding with the analyses. Phyloseq package (version 1.42.0) was used to calculate alpha and beta diversity (McMurdie and Holmes, 2013). Alpha diversity was estimated using the Observed Species, Chao1 (Chao, 1984), and abundance-based coverage estimator (ACE) indices (Chao and Lee, 1992). Beta diversity was analyzed using Bray–Curtis distances (Bray and Curtis, 1957) and visualized with nonmetric multidimensional scaling (nMDS); significance was assessed by permutational multivariate analysis of variance (PERMANOVA). Taxonomic groups with significant differences in abundance among different groups were identified by Linear Discriminant effect Size analysis (LEfSe) (Segata et al., 2011) using the microbiome Marker package (version 1.3.2) (Cao et al., 2022). Upsets plot showing how many ASVs and Species were unique and how many were shared between the groups was generated using UpSetR (version 1.4.0) (Nusrat et al., 2019) and ComplexUpset (version 1.3.3) (Krassowski et al., 2020; Lex et al., 2014). Rarefaction curves were generated using the MicrobiotaProcess package (version 1.6.6) (Xu et al., 2023). The aforementioned analyses were performed using R version 4.4.2.

## Results

### Nonmetric multidimensional scaling analysis of the *Drosophila melanogaster* microbial composition

**Figure 1:**
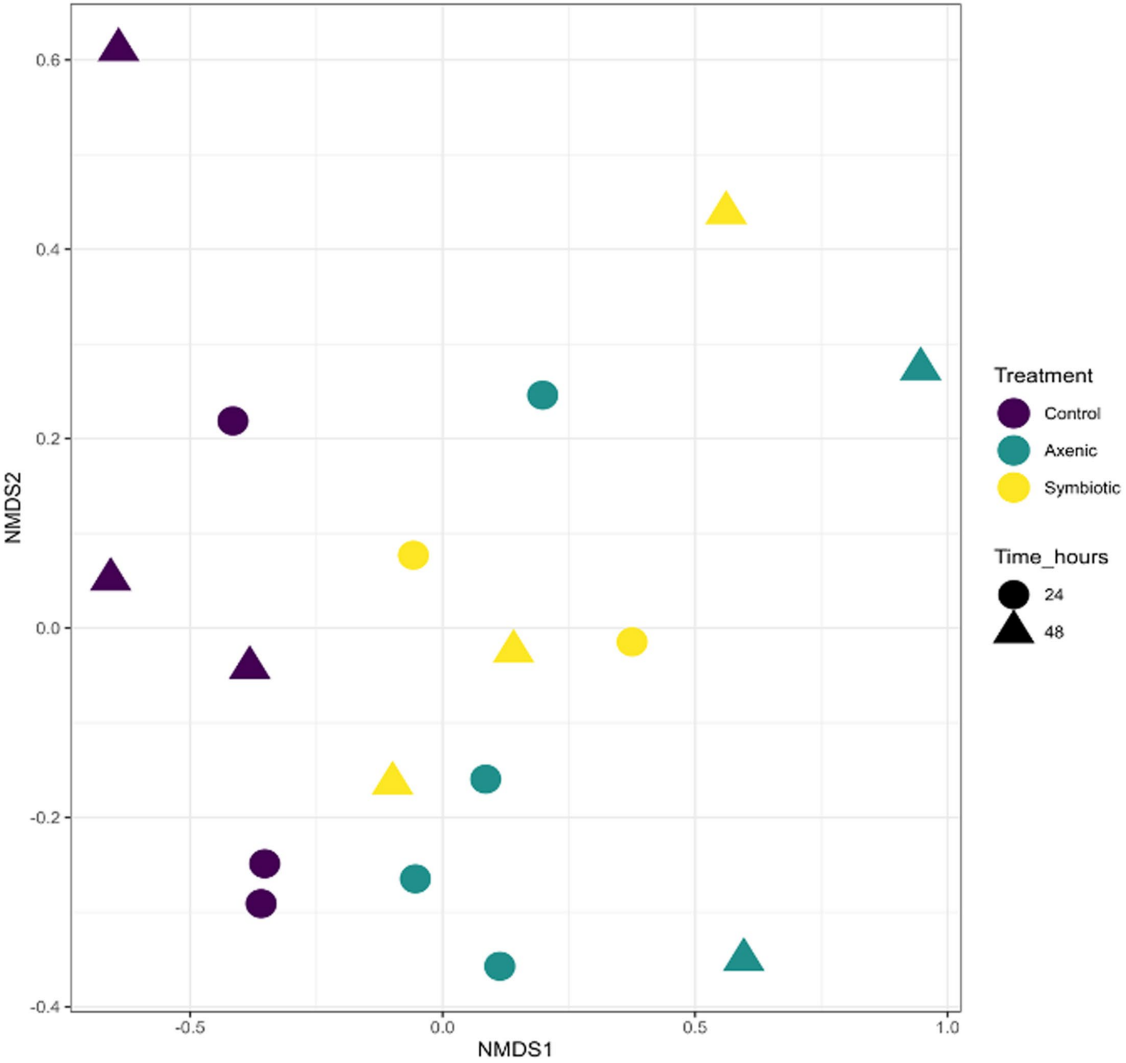
The Non-metric Multidimensional Scaling (NMDS) plot represents the similarity in microbial communities among different experimental groups: *Drosophila melanogaster* larvae infected with symbiotic *Heterorhabditis bacteriophora* (Symbiotic), *D. melanogaster* larvae infected with axenic *H. bacteriophora* (Axenic), and uninfected control *D. melanogaster* larvae. The samples were compared based on treatment (infection type), time points, and overall cluster.

Grouping the samples according to their infection status revealed significant statistical differences across the different treatments. This was confirmed by a PERMANOVA test, where an *R*^2^ value was 0.12 and a *p*-value was 0.044 (Figure 1). This indicates that there was meaningful variation between the infected and uninfected samples. However, when the samples were grouped based on the specific treatment conditions, *D. melanogaster* larvae infected with either symbiotic or axenic *H. bacteriophora* or larvae treated with water only (uninfected controls), there was no statistically significant difference between these subgroups (PERMANOVA: *R*^2^ = 0.18; *p*-value = 0.089). This suggests that the infection status is a primary differentiating factor, which means that it is the key variable that determined statistically significant changes in the microbial population in the fly larval microbiome. However, the type of infection - whether by symbiotic or axenic nematodes - or the absence of infection in control samples did not significantly impact the variation in microbial diversity. These findings were presented in the NMDS plot, where the samples are grouped into two distinct clusters- one for the uninfected control group and one for the infected group. The clusters reflect that the infection status had a stronger influence on the microbial composition of *D. melanogaster* larvae than the specific type of infection or the uninfected control condition.

### Unique and shared bacterial species in the *Drosophila melanogaster* larval microbiome in uninfected and nematode-infected treatments

**Figure 2:**
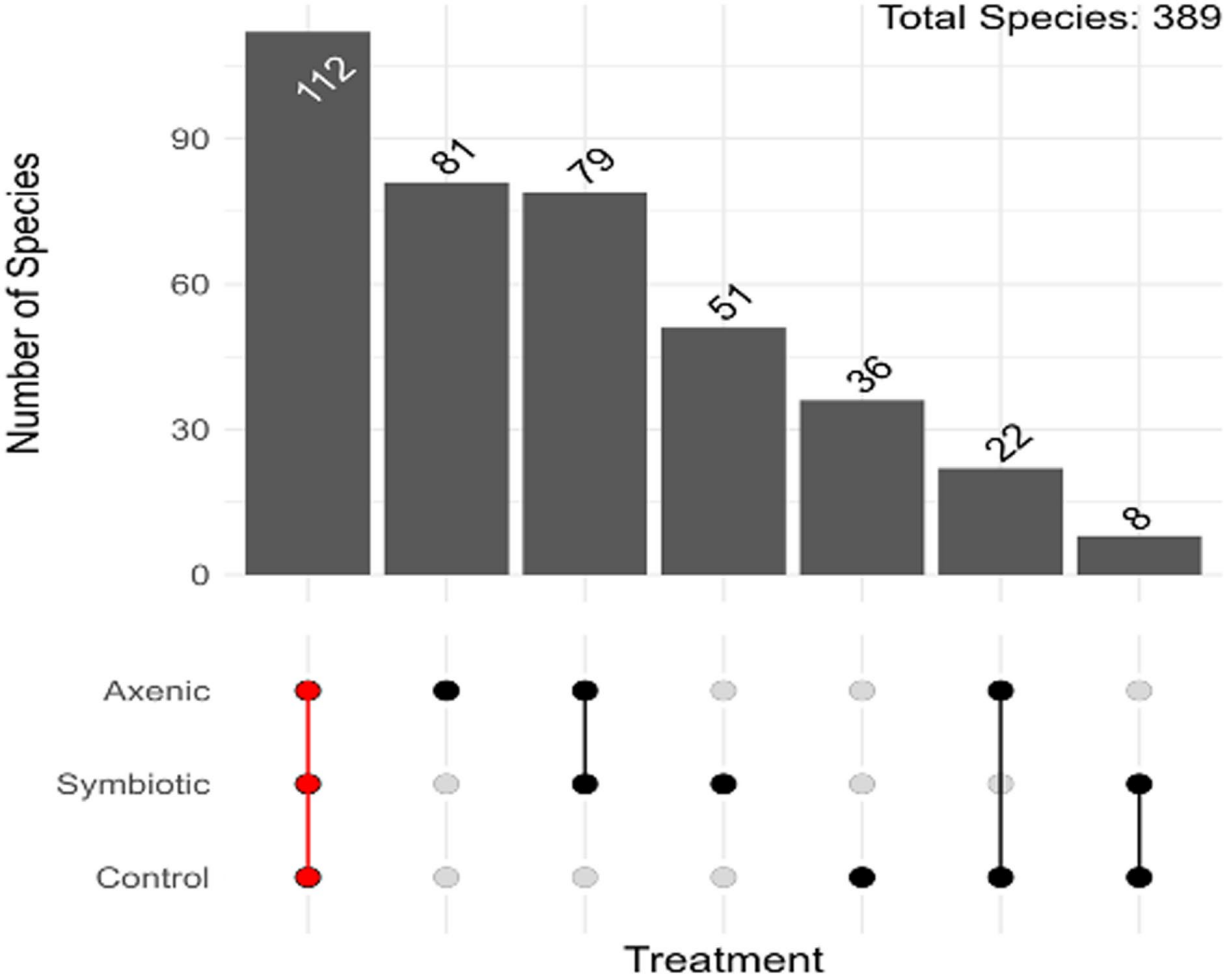
Distribution of the Amplicon Sequence Variants (ASVs) in axenic and symbiotic *Heterorhabditis bacteriophora* infection groups in *Drosophila melanogaster* larvae. The upset plot demonstrates the overlap and unique distribution of ASVs in *D. melanogaster* larvae infected with either axenic *H. bacteriophora* or symbiotic *H. bacteriophora* and in uninfected control larvae.

**Figure 3:**
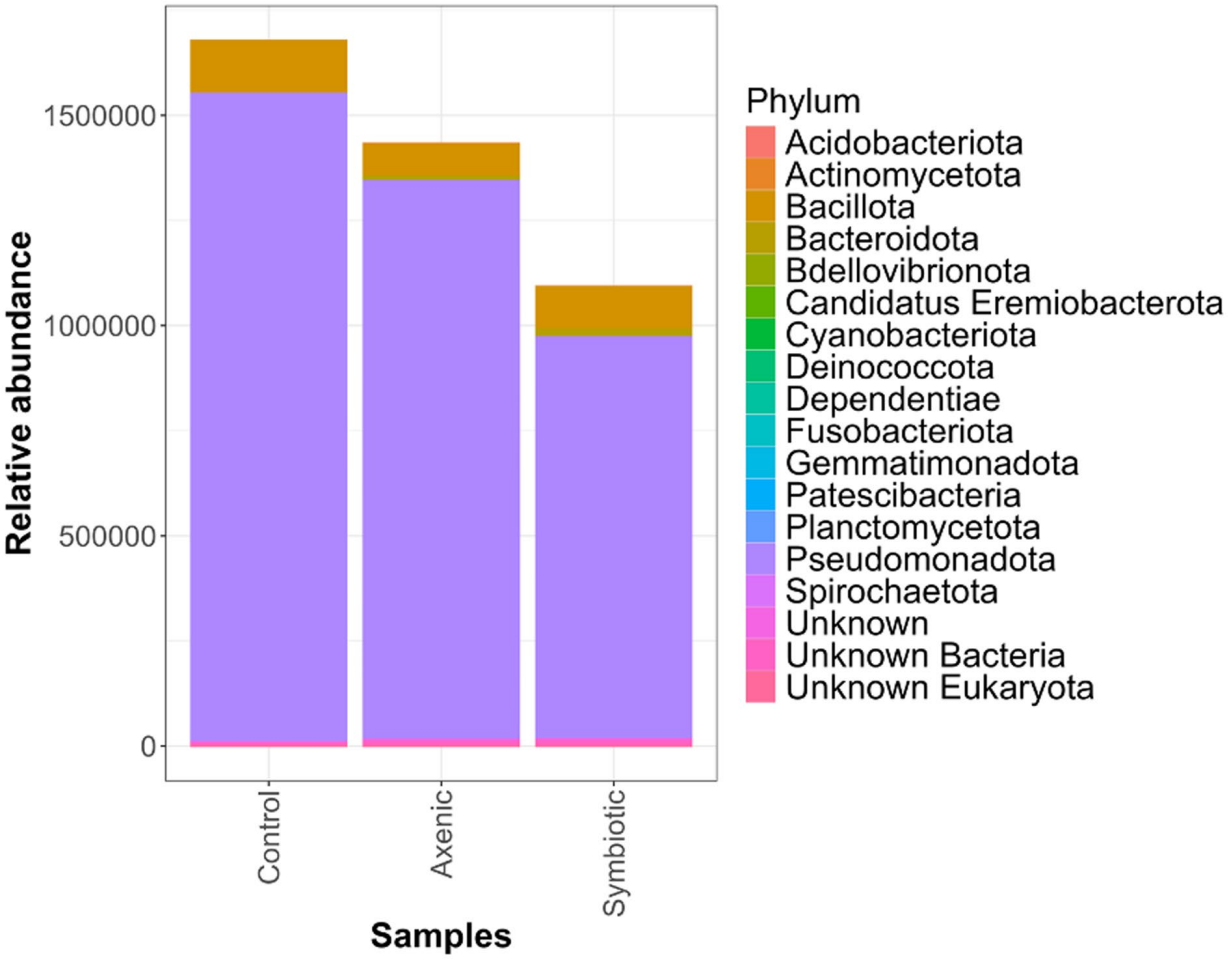
The relative abundance of the *Drosophila melanogaster* larval microbiome following infection with either axenic or symbiotic *Heterorhabditis bacteriophora* nematodes. The bar charts represent the total microbial composition (all phyla combined) across the three experimental groups: larvae infected with axenic *H. bacteriophora*, larvae infected with symbiotic *H. bacteriophora*, and control uninfected larvae treated with sterile water.

The Upset plot, which determines the unique and shared bacterial species among the three experimental groups (*D. melanogaster* larvae infected with either axenic or symbiotic *H. bacteriophora* and uninfected control larvae), shows that from the total of 389 bacterial species 115 were shared among the three treatments (Figure 2); the *D. melanogaster* larvae infected with axenic *H. bacteriophora* contain 68 unique bacterial species. As observed in Figure 3, the plot highlights how the mutualistic *P. luminescens* bacteria of *H. bacteriophora* impact the microbial community in the *D. melanogaster* larvae during infection with the symbiotic nematodes (nematode-bacteria complexes). Particularly, these results indicate that *D. melanogaster* larvae infected with symbiotic nematodes support a microbial community which is distinct from that found in larvae infected with axenic nematodes or that observed in uninfected individuals. As shown in Figure 3, this indicates that the presence of *P. luminescens* mutualistic bacteria in *H. bacteriophora* nematodes plays a pivotal role in shaping the microbial community and host-microbial environment during *D. melanogaster* larval infection.

### Impact of *Heterorhabditis bacteriophora* nematode infection on the abundance of bacterial taxa in the *Drosophila melanogaster* larval microbiome

We have found that the uninfected control larvae contain a higher relative abundance of certain bacterial taxa such as Pseudomonadota and Bacillota compared to larvae infected with either axenic or symbiotic *H. bacteriophora* (Figure 3). This result highlights the difference in the microbial profiles between the three experimental groups. However, despite the differences, the bacterial communities observed in both uninfected controls and nematode-infected larvae mainly consisted of Pseudomonadota bacteria, namely from the Alphaproteobacteria and Gammaproteobacteria classes. The abundance of Pseudomonadota across all experimental groups suggests that these bacteria are characterizing the core microbiome of *D. melanogaster* larvae, regardless of their nematode infection status.

### Differentially abundant bacterial species found in uninfected and nematode infected *Drosophila melanogaster* larval microbiome

**Figure 4:**
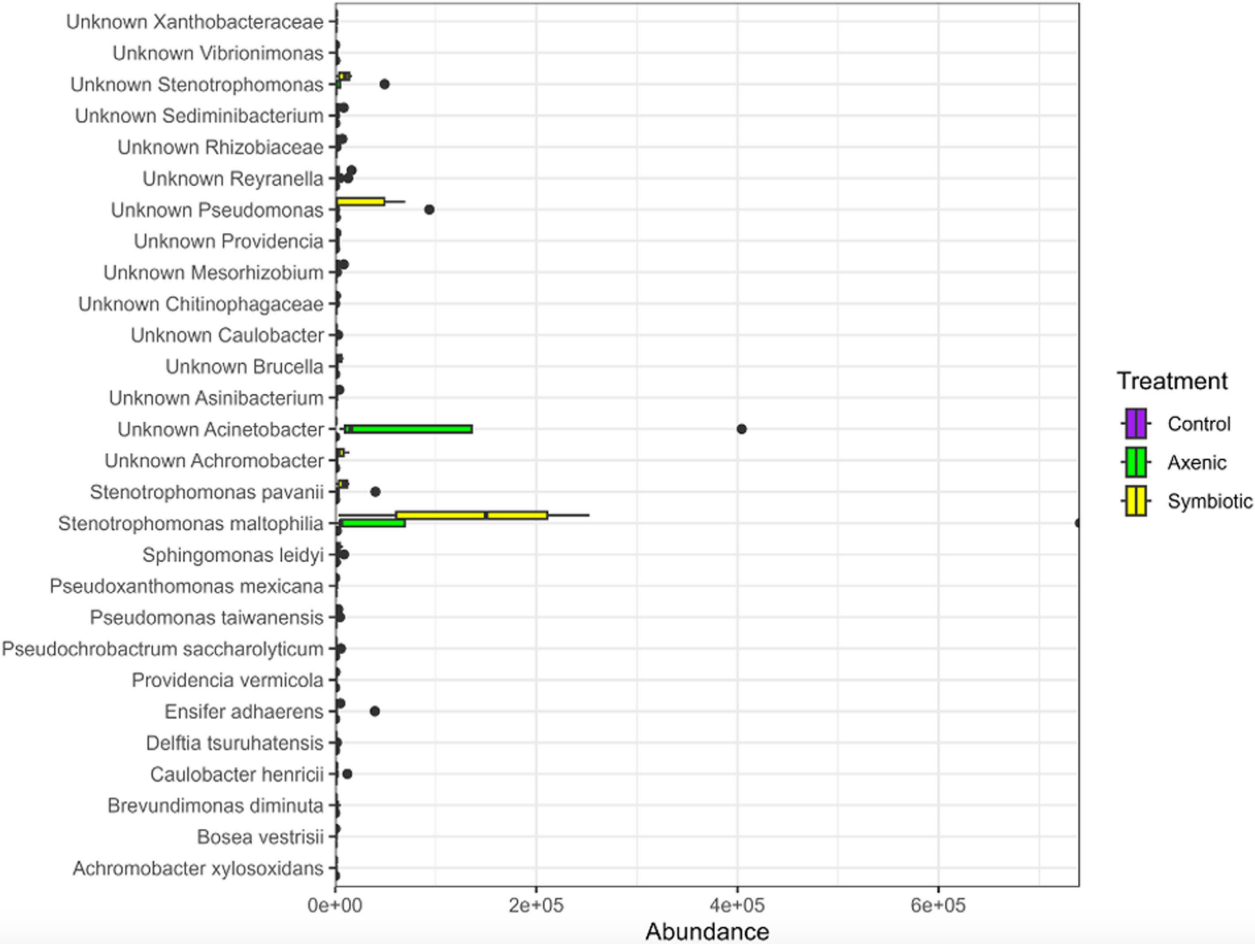
The LEfSe plot demonstrates differentially abundant bacterial species across parasitic nematode infection groups within the *Drosophila melanogaster* larval microbiome. It compares the relative abundance of bacterial species in *D. melanogaster* following infection with either *Heterorhabditis bacteriophora* symbiotic or axenic nematodes with the abundance of bacterial species in the uninfected control group.

Linear Discriminant Analysis Effect Size (LEfSe) was performed using the three treatment groups (infection with *H. bacteriophora* symbiotic or axenic nematodes, or uninfected larvae) as a Group, while the two time points were clustered together as a Subgroup (Figure 4). The analysis showed a total of 28 bacterial species that were differentially abundant among the treatments. While the larvae infected with *H. bacteriophora* axenic nematodes showed 18 significantly enriched biomarkers, 10 biomarkers were more abundant in larvae infected with *H. bacteriophora* symbiotic nematodes. *Stenotrophomonas maltophilia*, one of the identified bacterial species, was highly abundant in larvae infected with *H. bacteriophora* axenic nematodes compared to the other treatments. From this analysis, it could be inferred that the presence or absence of the symbiotic bacteria *P. luminescens* influences the microbial composition of *D. melanogaster* larvae during nematode infection. Particularly, axenic *H. bacteriophora* nematodes lead to a greater shift in bacterial population diversity and are enriched in certain taxa such as *Stenotrophomonas maltophilia.* This suggests strong affirmation that the symbiotic bacteria play a key role in modulating the fly larval microbiome.

### Shifts in *Drosophila melanogaster* larval microbiome during *Heterorhabditis bacteriophora* nematode infection

**Figure 5:**
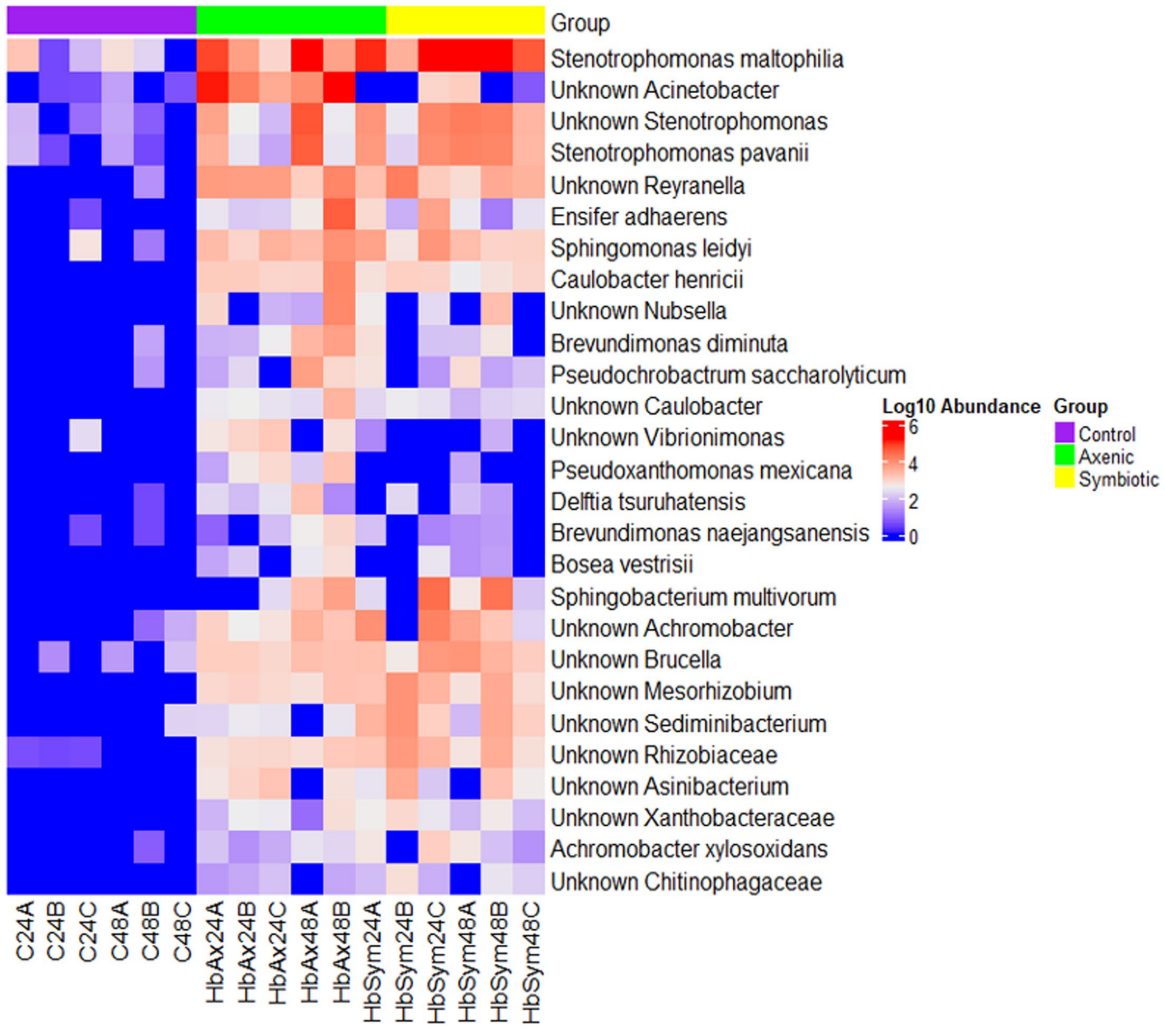
The effect of symbiotic and axenic *Heterorhabditis bacteriophora* nematode infection on the *Drosophila melanogaster* larval microbiome composition. The heatmap illustrates differentially abundant bacterial species in the *D. melanogaster* larval microbiome in the control uninfected larvae, the *H. bacteriophora* symbiotic nematode-infected larvae, and the *H. bacteriophora* axenic nematode-infected larvae at 24- and 48-h post-infection. The color scale on the right represents Log10 abundance, with red indicating higher abundance and blue indicating lower abundance.

When plotted on a heatmap, the results of LefSe analysis show the distribution of the 28 bacterial species in the *D. melanogaster* larval microbiome in the uninfected control samples and the samples of the two groups infected with either symbiotic or axenic *H. bacteriophora* nematodes at 24 and 48 h (Figure 5). A noticeable increase in the bacterial abundance in the fly larval microbiome was observed in both nematode-infected groups (symbiotic or axenic *H. bacteriophora*-infected groups) in comparison to the uninfected control group, with the most predominant being *Stenotrophomonas maltophilia*, *Reyranella*, *Ensifer adhaerens*, *Sphingomonas leidyi*, *Caulobacter henricii*, *Sphingobacterium multivorum*, *Achromobacter*, *Brucella*, *Mesorhizobiun*, *Sediminibacterium*, *Rhizobiaceae*, Xanthobacteraceae, and Chitinophagaceae. Between the two nematode infected groups, a higher abundance of *Nubsella*, *Brevudimonas diminuta*, *Vibrionimonas*, *Pseudoxanthomonas mexicana*, *Delftia tsuruhatensis*, *Brevundimonas naejangsanensis*, and *Bosea vestrisii* was observed in the larvae infected with axenic *H. bacteriophora*; the larvae infected with symbiotic *H. bacteriophora* nematodes seemed to be closer to the control samples. Within the axenic *H. bacteriophora* infection treatment, almost all bacteria found 24 h following infection were also found in the similar abundance at 48 h. *Stenotrophomonas maltophilia* was the species with the highest relative abundance in larvae infected with axenic *H. bacteriophora*, exhibiting a 5-fold (log10) increase compared to the uninfected control group.

### Differences in the microbial communities in nematode-infected and uninfected treatment groups in the *Drosophila melanogaster* larval microbiome

**Figure 6:**
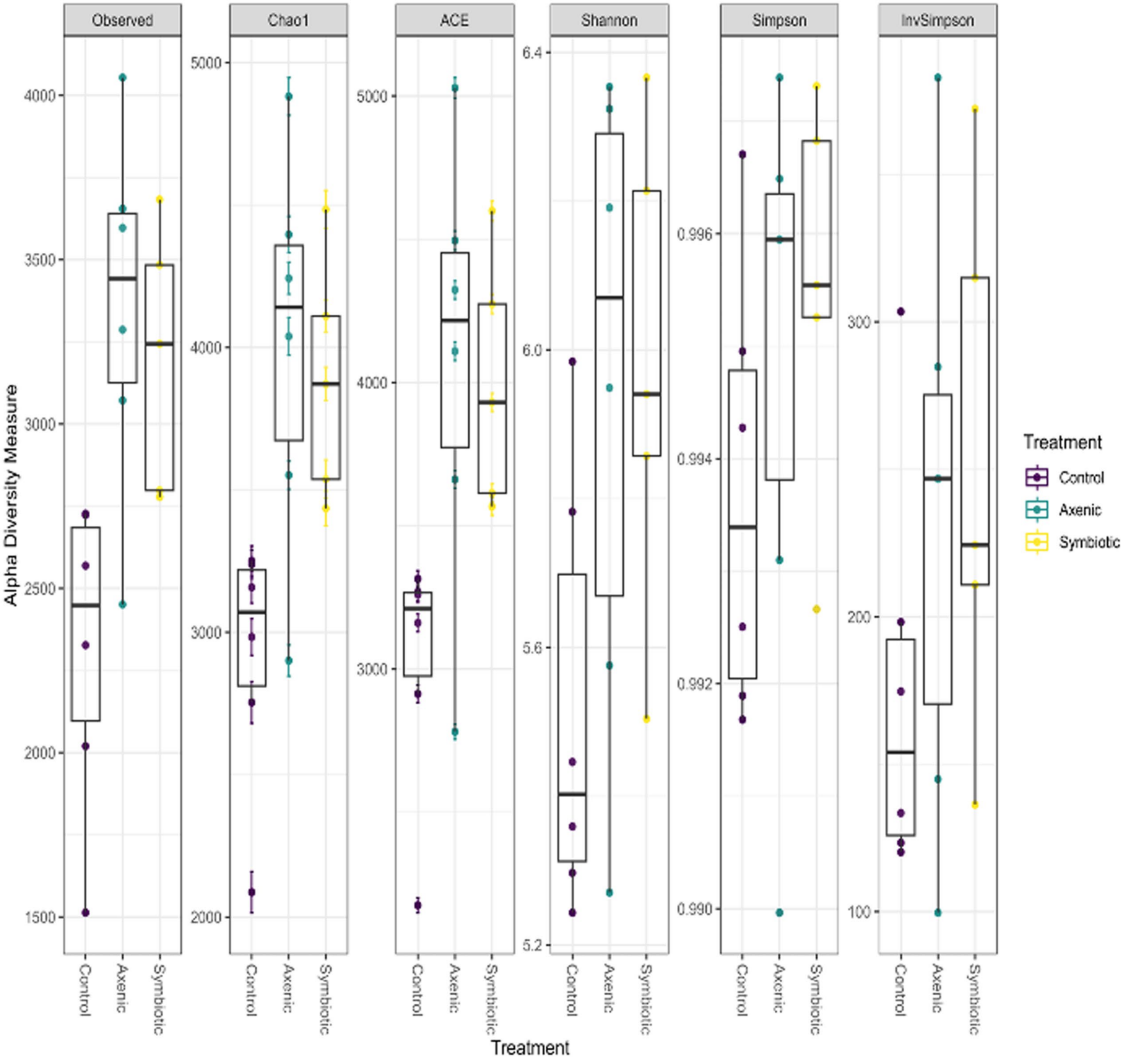
The boxplots show the distribution of the alpha diversity measures, including the observed number of Amplicon Sequence Variants (ASVs), Chao1, and ACE indices, to estimate the microbial population diversity across different treatment groups. The data represent the microbial communities of *Drosophila melanogaster* larvae infected with either axenic or symbiotic *Heterorhabditis bacteriophora* nematodes.

Boxplots show the alpha diversity indices, such as the observed number of Amplicon Sequence Variants (ASVs), Chao1, and Abundance-based Coverage Estimator (ACE), which estimate the richness and diversity of microbial communities in the *D. melanogaster* larvae upon infection with either axenic or symbiotic *H. bacteriophora* (Figure 6). In terms of the number of ASVs and the other alpha diversity metrics, the boxplots show similar values for both types of nematode-infected larvae. This indicates that the overall microbial diversity of the *D. melanogaster* microbiome was similar regardless of the type of nematode infection (symbiotic *H. bacteriophora* containing the symbiotic bacteria *P. luminescens* and axenic nematodes without the symbiotic bacteria). The higher values of the alpha diversity indices recorded in the EPN-infected groups compared to the uninfected control group indicate that the microbial communities in the infected (either by symbiotic or axenic *H. bacteriophora*) larvae were more diverse, i.e., that the larval infection results in higher alpha diversities.

## Discussion

Our findings demonstrate the significant impact of *H. bacteriophora* infection on the microbial composition of *D. melanogaster* larvae, with unique shifts observed between the symbiotic and axenic nematode infections. The outcomes of this research were evidenced by the nMDS plot and confirmed by a PERMANOVA test, which highlighted the nematode infection as the primary differentiating factor. However, the nematode treatment type—whether the fly larvae were infected with symbiotic or axenic *H. bacteriophora*—did not significantly affect microbial diversity, as indicated by a non-significant PERMANOVA result. These findings suggest that nematode infection, rather than the type of nematode (symbiotic or axenic), plays a key role in shaping the *D. melanogaster* larval microbiome upon infection with the EPN *H. bacteriophora*.

When analyzing differentially abundant bacterial species using LEfSe, we identified 28 bacterial strains across the three treatment groups, with a remarkably high abundance of *S. maltophilia* observed in the axenic *H. bacteriophora* infection group (Fierst et al., 2017; Brooke, 2012). This potentially suggests an ecological disruption in the absence of the symbiotic bacteria *P. luminescens*, also shown by the heatmap representation of the LefSe results. This supports the hypothesis that EPN infection alters the microbial landscape in *D. melanogaster* larvae. Overall, the microbial community of the fly larvae was predominantly composed of *Pseudomonadota* bacteria which were present in all experimental groups, indicating that this phylum plays a fundamental role in the larval microbiome, regardless of the infection status; however, specific differences were identified among the distribution of Gammaproteobacteria which were more diverse in the infected samples compared to the control samples. Additionally, a comparison of the alpha diversity indices (ASVs, Chao1, ACE) showed higher microbial diversity in the EPN-infected groups than in the uninfected controls, implying a change in microbial richness in *D. melanogaster* larvae during parasitic nematode infection. Moreover, the analysis of unique and shared bacterial species highlighted that axenic *H. bacteriophora* infection resulted in the occurrence of a similar number of unique bacterial species compared to symbiotic *H. bacteriophora* infection; interestingly, the uninfected treatment (control) was characterized by only 36 unique species. This finding suggests that the absence of the mutualistic bacteria *P. luminescens* may create ecological niches that allow other microbes to thrive. Together, these observations underscore the importance of EPN infection in shaping the microbial flora in *D. melanogaster* larvae, with axenic and symbiotic *H. bacteriophora* nematodes exerting different influences on the host microbiome.

Recent work has increasingly focused on the gut microbiome and its role in immunity. The insect gut microbiome is instrumental in regulating the immune response by altering the pH and gastric enzyme levels, vigorously competing with pathogens for resources such as nutrients and habitat and antimicrobial substances that can combat invading pathogens (Bai et al., 2021). The comparatively simple microbiome of *D. melanogaster* offers beneficial insights into the function of the mammalian microbiome. The fruit fly gut is home to various microorganisms interacting positively with the host, including mutualistic and commensal relationships. These interactions play a significant role in maintaining a healthy microbial environment that benefits the host and the microbial community (Tafesh-Edwards and Eleftherianos, 2023).

During parasitic invasions, the host gut responds to invading pathogens with higher levels of immune system activation stimulated by pathogenic bacteria. In mammals and *D. melanogaster*, immune signaling pathways such as JAK–STAT, NF-κB, and Hippo regulate tissue repair, stem cell proliferation, and immune cell activities to maintain homeostasis in the intestinal tract. While the host microbiome plays a significant role in maintaining this balance, pathogenic infections can trigger immune responses that reveal a complex interaction between the microbial community and the host immune system (Liu et al., 2017).

In our study, we observed higher microbial diversity in the EPN-infected *D. melanogaster* larvae compared to the uninfected controls, as evidenced by the alpha-diversity metrics, which include the observed number of ASVs, Chao1, and ACE. This indicates that parasitic nematode infections, regardless of whether the infection was due to symbiotic or axenic *H. bacteriophora*, showed an increase in the microbial population and its complexity. The observed increase in diversity may hint ecological shifts within the microbial flora as a result of EPN infection. Moreover, upon further investigation between the symbiotic versus axenic *H. bacteriophora* infections, we observed that the presence of *P. luminescens* in the symbiotic *H. bacteriophora* nematodes limits the proliferation of certain bacterial taxa, such as *S. maltophilia*, which exhibited higher abundance in the axenic *H. bacteriophora*-infected fly larval group; however, it should be noted that *S. maltophilia* was present in both types of infection, therefore it is characterizing *H. bacteriophora* nematodes. The above suggest that *P. luminescens* may play a crucial role in managing the microbial community by restricting the growth of specific bacterial species in the host gut. However, axenic nematode infections create a favorable environment with fewer constraints, allowing a more diverse microbial population to thrive in the larval gut.

Despite the significant changes in the microbiome related to EPN infection, the bacterial phylum *Pseudomonadota*, and in particular the class Alphaproteobacteria, persistently dominated the *D. melanogaster* larval microbiome across all experimental groups, including both uninfected and infected larvae. This suggests that *Pseudomonadota* plays a critical, possibly indispensable, role in the microbial population within the fruit fly gut. The dominance of this phylum is remarkable even in the presence of parasitic nematode infection, regardless of whether the larvae were exposed to symbiotic or axenic *H. bacteriophora*. This indicates that these bacteria taxa might be involved in foundational ecological or physiological functions that are crucial for maintaining microbial homeostasis in the fly larvae. *Pseudomonadota* has been observed to dominate the gut microbiomes of various insect species, such as *Bombus* bumblebees, *Cephalotes* ants, and various termite species (Suenami et al., 2023). In the context of infection with either symbiotic or axenic *H. bacteriophora*, the persistence of *Pseudomonadota* implies that these bacteria may contribute to a stable microbiome that could mitigate the damage caused by parasitic nematode invasion. Therefore, the *Pseudomonadota* phylum may play a pivotal role in stabilizing the larval microbiome and promoting the health of the host even under adverse conditions.

Our results further emphasize the existence of a core *D. melanogaster* larval microbiome comprising 389 bacterial species, out of which 115 are shared across all experimental groups, including uninfected larvae used as controls and those infected with either symbiotic or axenic *H. bacteriophora*. This core microbial community potentially contributes to maintaining the stability and functionality of the fly larval microbiome, regardless of infection status. This disruption or shift could indicate a compensatory microbial population during axenic *H. bacteriophora* infection. The influence of *P. luminescens* fosters a more stable and less diverse microbiome. Our findings further support the hypothesis that *P. luminescens* not only aids nematode virulence during symbiotic *H. bacteriophora* infection in *D. melanogaster* but also contributes to shaping the overall host larval microbiome.

### Future directions and concluding remarks

Future research should investigate the unresolved questions regarding the shift of the microbial community on the host immune system response and overall survival. Notably, exploring how the altered microbial composition in *D. melanogaster* larvae following EPN infection impacts the host immune activity, metabolic function and behavioral responses could provide essential insights into host–parasite interactions. Also, the effect of EPN infection on the mucosal immune reaction of *D. melanogaster* or other insects that might, in turn, shape the microbiota as well as the insect host mucosal responses that might interfere with the endogenous microbiota will form future research objectives. The current research focused on two time points, 24- and 48-h post-infection. Future studies could expand this approach to include additional time points to better understand how microbial communities evolve and their consequent impact on the host. Moreover, multiple species of parasitic nematodes could be incorporated into future research to understand how different EPN-host–microbe interactions stimulate microbial community shifts. Assessing various environmental parameters, such as temperature, could reveal how these factors influence the interactions between nematode infection and host-microbiome composition, which would propose a more comprehensive understanding of the ecological and environmental impact on host-microbial community dynamics.

Furthermore, the application of advanced high-throughput sequencing technologies could facilitate the identification of unique microbial taxa responsible for altering immune responses. This could lead to the innovation of novel therapeutic strategies that would target microbial communities. Integrating approaches such as transcriptomics and metabolomics could also provide profound insights into the molecular mechanisms between the host, the EPNs, and the microbial population. This would pave the way for improved strategies in managing parasitic nematode infections in agriculture and medicine. In conclusion, by expanding our knowledge of these complex relationships, future research can benefit from practical solutions to combat parasitic infections and improve human health.

## Data Availability

All the raw sequence files of this study were submitted to the European Nucleotide Archive (ENA) with the study accession number PRJEB85826. Further inquiries can be directed to the corresponding author.

## References

[ref1] Bai S. Yao Z. Raza M. F. Cai Z. Zhang H. (2021). Regulatory mechanisms of microbial homeostasis in insect gut. Insect Sci. 28, 286–301. doi: 10.1111/1744-7917.12868, PMID: 32888254

[ref2] Bastin A. Eleftherianos I. (2023). Heterorhabditis bacteriophora. Trends Parasitol. 39, 603–604. doi: 10.1016/j.pt.2023.04.006, PMID: 37188598

[ref3] Belkaid Y. Harrison O. J. (2017). Homeostatic immunity and the microbiota. Immunity 46, 562–576. doi: 10.1016/j.immuni.2017.04.008, PMID: 28423337 PMC5604871

[ref4] Bray J. R. Curtis J. T. (1957). An ordination of the upland forest communities of southern Wisconsin. Ecol. Monogr. 27, 326–349. doi: 10.2307/1942268

[ref5] Brooke J. S. (2012). *Stenotrophomonas maltophilia*: an emerging global opportunistic pathogen. Clin. Microbiol. Rev. 25, 2–41. doi: 10.1128/CMR.00019-11, PMID: 22232370 PMC3255966

[ref6] Cao Y. Dong Q. Wang D. Zhang P. Liu Y. Niu C. (2022). microbiomeMarker: an R/Bioconductor package for microbiome marker identification and visualization. Bioinformatics 38, 4027–4029. doi: 10.1093/bioinformatics/btac438, PMID: 35771644

[ref7] Capo F. Wilson A. Di Cara F. (2019). The intestine of *Drosophila melanogaster*: an emerging versatile model system to study intestinal epithelial homeostasis and host-microbial interactions in humans. Microorganisms 7:336. doi: 10.3390/microorganisms7090336, PMID: 31505811 PMC6780840

[ref8] Castillo J. C. Shokal U. Eleftherianos I. (2012). A novel method for infecting *Drosophila* adult flies with insect pathogenic nematodes. Virulence 3, 339–347. doi: 10.4161/viru.20244, PMID: 22546901 PMC3442847

[ref9] Chao A. (1984). Non-parametric estimation of the classes in a population. Scand. J. Stat. 11, 265–270. doi: 10.2307/4615964

[ref10] Chao A. Lee S.-M. (1992). Estimating the number of classes via sample coverage. J. Am. Stat. Assoc. 87, 210–217. doi: 10.1080/01621459.1992.10475194

[ref11] Douglas A. E. (2018). The *Drosophila* model for microbiome research. Lab. Anim. 47, 157–164. doi: 10.1038/s41684-018-0065-0, PMID: 29795158 PMC6586217

[ref12] Eleftherianos I. ffrench-Constant R. H. Clarke D. J. Dowling A. J. Reynolds S. E. (2010). Dissecting the immune response to the entomopathogen *Photorhabdus*. Trends Microbiol. 18, 552–560. doi: 10.1016/j.tim.2010.09.006, PMID: 21035345

[ref13] Fierst J. L. Murdock D. A. Thanthiriwatte C. Willis J. H. Phillips P. C. (2017). Metagenome-assembled draft genome sequence of a novel microbial *Stenotrophomonas maltophilia* strain isolated from *Caenorhabditis remanei* tissue. Genome Announc. 5:e01646–16. doi: 10.1128/genomeA.01646-16, PMID: 28209833 PMC5313625

[ref14] Heryanto C. Ratnappan R. O’Halloran D. M. Hawdon J. M. Eleftherianos I. (2022). Culturing and genetically manipulating entomopathogenic nematodes. J. Vis. Exp.:e63885. doi: 10.3791/63885 35435903

[ref15] Kenney E. Hawdon J. M. O'Halloran D. M. Eleftherianos I. (2021). Secreted virulence factors from *Heterorhabditis bacteriophora* highlight its utility as a model parasite among clade V nematodes. Int. J. Parasitol. 51, 321–325. doi: 10.1016/j.ijpara.2020.10.004, PMID: 33421438

[ref16] Krassowski M. Das V. Sahu S. K. Misra B. B. (2020). State of the field in multi-omics research: from computational needs to data mining and sharing. Front. Genet. 11:610798. doi: 10.3389/fgene.2020.610798, PMID: 33362867 PMC7758509

[ref17] Lemaitre B. Hoffmann J. (2007). The host defense of *Drosophila melanogaster*. Annu. Rev. Immunol. 25, 697–743. doi: 10.1146/annurev.immunol.25.022106.141615, PMID: 17201680

[ref18] Lex A. Gehlenborg N. Strobelt H. Vuillemot R. Pfister H. (2014). UpSet: visualization of intersecting sets. IEEE Trans. Vis. Comput. Graph. 20, 1983–1992. doi: 10.1109/TVCG.2014.2346248, PMID: 26356912 PMC4720993

[ref19] Liu X. Hodgson J. J. Buchon N. (2017). *Drosophila* as a model for homeostatic, antibacterial, and antiviral mechanisms in the gut. PLoS Pathog. 13:e1006277. doi: 10.1371/journal.ppat.1006277, PMID: 28472194 PMC5417715

[ref20] McLean F. Berger D. Laetsch D. R. Schwartz H. T. Blaxter M. (2018). Improving the annotation of the *Heterorhabditis bacteriophora* genome. GigaScience 7:giy034. doi: 10.1093/gigascience/giy034, PMID: 29617768 PMC5906903

[ref21] McMurdie P. J. Holmes S. (2013). Phyloseq: an R package for reproducible interactive analysis and graphics of microbiome census data. PLoS One 8:e61217. doi: 10.1371/journal.pone.0061217, PMID: 23630581 PMC3632530

[ref22] Noguez J. H. Conner E. S. Zhou Y. Ciche T. A. Ragains J. R. Butcher R. A. (2012). A novel ascaroside controls the parasitic life cycle of the entomopathogenic nematode *Heterorhabditis bacteriophora*. ACS Chem. Biol. 7, 961–966. doi: 10.1021/cb300056q, PMID: 22444073 PMC3548670

[ref23] Nusrat S. Harbig T. Gehlenborg N. (2019). Tasks, techniques, and tools for genomic data visualization. Comput. Graph. Forum 38, 781–805. doi: 10.1111/cgf.13727, PMID: 31768085 PMC6876635

[ref24] O'Cathail C. Ahamed A. Burgin J. Cummins C. Devaraj R. Gueye K.. (2025). The European nucleotide archive in 20204. Nucleic Acids Res. 53, D49–D55. doi: 10.1093/nar/gkae975 39558171 PMC11701661

[ref25] Ozakman Y. Eleftherianos I. (2021). Nematode infection and antinematode immunity in *Drosophila*. Trends Parasitol. 37, 1002–1013. doi: 10.1016/j.pt.2021.06.001, PMID: 34154933

[ref26] Ozakman Y. Raval D. Eleftherianos I. (2021). Activin and BMP signaling activity affects different aspects of host anti-nematode immunity in *Drosophila melanogaster*. Front. Immunol. 12:795331. doi: 10.3389/fimmu.2021.795331, PMID: 35003118 PMC8727596

[ref27] Quast C. Pruesse E. Yilmaz P. Gerken J. Schweer T. Yarza P.. (2013). The SILVA ribosomal RNA gene database project: improved data processing and web-based tools. Nucleic Acids Res. 41, D590–D596. doi: 10.1093/nar/gks1219 23193283 PMC3531112

[ref28] Reiter L. T. Potocki L. Chien S. Gribskov M. Bier E. (2001). A systematic analysis of human disease-associated gene sequences in *Drosophila melanogaster*. Genome Res. 11, 1114–1125. doi: 10.1101/gr.169101, PMID: 11381037 PMC311089

[ref29] Segata N. Izard J. Waldron L. Gevers D. Miropolsky L. Garrett W. S.. (2011). Metagenomic biomarker discovery and explanation. Genome Biol. 12:R60. doi: 10.1186/gb-2011-12-6-r60, PMID: 21702898 PMC3218848

[ref30] Suenami S. Koto A. Miyazaki R. (2023). Basic structures of gut bacterial communities in eusocial insects. Insects 14:444. doi: 10.3390/insects14050444, PMID: 37233072 PMC10231122

[ref31] Tafesh-Edwards G. Eleftherianos I. (2023). The role of *Drosophila* microbiota in gut homeostasis and immunity. Gut Microbes 15:2208503. doi: 10.1080/19490976.2023.2208503, PMID: 37129195 PMC10155630

[ref32] Tarasco E. Fanelli E. Salvemini C. El-Khoury Y. Troccoli A. Vovlas A.. (2023). Entomopathogenic nematodes and their symbiotic bacteria: from genes to field uses. Front. Insect Sci. 3:1195254. doi: 10.3389/finsc.2023.1195254, PMID: 38469514 PMC10926393

[ref33] Waterfield N. R. Ciche T. Clarke D. (2009). *Photorhabdus* and a host of hosts. Ann. Rev. Microbiol. 63, 557–574. doi: 10.1146/annurev.micro.091208.073507, PMID: 19575559

[ref34] Wiertsema S. P. van Bergenhenegouwen J. Garssen J. Knippels L. M. J. (2021). The interplay between the gut microbiome and the immune system in the context of infectious diseases throughout life and the role of nutrition in optimizing treatment strategies. Nutrients 13:886. doi: 10.3390/nu13030886, PMID: 33803407 PMC8001875

[ref35] Xu S. Zhan L. Tang W. Wang Q. Dai Z. Zhou L.. (2023). MicrobiotaProcess: a comprehensive R package for deep mining microbiome. The Innovation 4:100388. doi: 10.1016/j.xinn.2023.100388, PMID: 36895758 PMC9988672

